# Podocytopathy and Glomerular Basement Membrane Anomalies in Two Patients With Cubilin Gene Mutations

**DOI:** 10.7759/cureus.34730

**Published:** 2023-02-07

**Authors:** Sara Madureira Gomes, Ana Isabel Igreja, Roberto Silva, João Paulo Oliveira, Helena Pinto

**Affiliations:** 1 Department of Pediatrics, Centro Hospitalar Universitário São João, Porto, PRT; 2 Department of Pediatrics, Centro Hospitalar De Trás-Os-Montes E Alto Douro, Vila Real, PRT; 3 Department of Pathology, Centro Hospitalar Universitário São João, Porto, PRT; 4 Faculty of Medicine, University of Porto, Porto, PRT; 5 Department of Genetics, Centro Hospitalar Universitário São João, Porto, PRT; 6 Institute for Research and Innovation in Health, University of Porto, Porto, PRT; 7 Pediatric Nephrology Unit, Centro Hospitalar Universitário São João, Porto, PRT

**Keywords:** pediatrics, glomerular basement membrane, podocytopathy, cubilin, proteinuria

## Abstract

Proteinuria is a frequent finding in pediatric patients and in most cases, it is intermittent or transient. When proteinuria is moderate/severe and persistent, it may require an extensive complementary study, histopathological examination and genetic test, in order to clarify its etiology. Cubilin (CUBN) is a large glycosylated extracellular protein, initially detected in proximal tubular cells, and later in podocytes. Isolated persistent proteinuria caused by cubilin gene mutations is rare, only a few cases have been reported in the literature and even fewer patients underwent renal biopsy and electron microscopy that could help to elucidate the pathogenesis of the disease.

The authors describe two pediatric clinical cases referred to pediatric nephrology consultation due to persistent proteinuria. Neither of them had any other complaints, and renal function and immunological and serological studies were normal. Renal histopathology showed podocytes changes and glomerular basal membrane alterations suggestive of Alport Syndrome. The genetic study identified two heterozygous variants in the cubilin gene in both, also later identified in their parents. They were started on ramipril, with improvement in proteinuria, and both patients remain asymptomatic and without changes in renal function.

At present, due to the uncertainty of prognosis, it is suggested to keep CUBN gene mutation patients under close surveillance of proteinuria and renal function. The variable ultrastructural patterns of podocytopathy and glomerular basal membrane alterations in kidney biopsies of pediatric patients with proteinuria should lead to the diagnostic possibility of CUBN gene mutation in the differential diagnosis.

## Introduction

Proteinuria is defined as the presence of non-physiological amounts of protein in the urine [1]. It is a frequent finding in pediatric patients, in most cases, intermittent or transient. However when it is moderate/severe and persistent, it may require an extensive complementary study, histopathological examination and genetic test, in order to clarify its etiology [2].

Cubilin (CUBN) is a large glycosylated extracellular protein, first identified as the vitamin B12/intrinsic factor complex receptor of the ileal mucosa. In the kidney, it was initially detected in proximal tubular cells [3-5], and later in podocytes [4,6]. It acts as ligand-binding site for albumin, intrinsic factor-B12 complex, vitamin carrier proteins, lipoproteins and other proteins, promoting the endocytosis of these ligands. The majority of albumin in primary urine (approximately 3g per day) is reabsorbed through cubilin-mediated endocytosis, resulting in very small amount of albumin in the final urine [3].

Isolated persistent proteinuria caused by cubilin gene mutations is rare, only a few cases have been reported in the literature and even fewer patients underwent renal biopsy and electron microscopy that could help to elucidate the pathogenesis of the disease [6].

The authors describe two pediatric clinical cases with isolated persistent proteinuria due to CUBN gene mutations and similar changes in renal biopsy.

## Case presentation

Case 1

A 7-year-old boy was referred to the pediatric nephrology consultation for persistent nephrotic proteinuria (maximum protein/creatinine ratio 2.7), diagnosed in the context of acute gastroenteritis. He was also diagnosed with congenital ichthyosis and his maternal grandfather died of end-stage renal disease of unknown cause. He had no edema, hypertension, hematuria, deafness or ocular abnormalities. Urinalysis did not show hematuria, glycosuria or abnormal electrolytes indexes. Renal function (urea 30 mg/dL; creatinine 0.36 mg/dL), serum electrolytes, albumin (41.3 g/L) and total serum protein (63.7 g/L) were normal. There was neither anemia (Hb 13.7 g/dL) nor changes in lipid profile. The immunological study was normal (IgA, IgG, IgM, C3c, C4, antinuclear antibodies (ANA), antibodies to double-stranded DNA (anti-ds-DNA), anti-GMB antibodies, anticardiolipin antibodies, anti-ENA antibodies). Serological (human immunodeficiency virus (HIV), hepatitis C virus (HCV), hepatitis B virus (HBV)) studies performed were unremarkable. Renal ultrasound revealed no changes.

**Figure 1:**
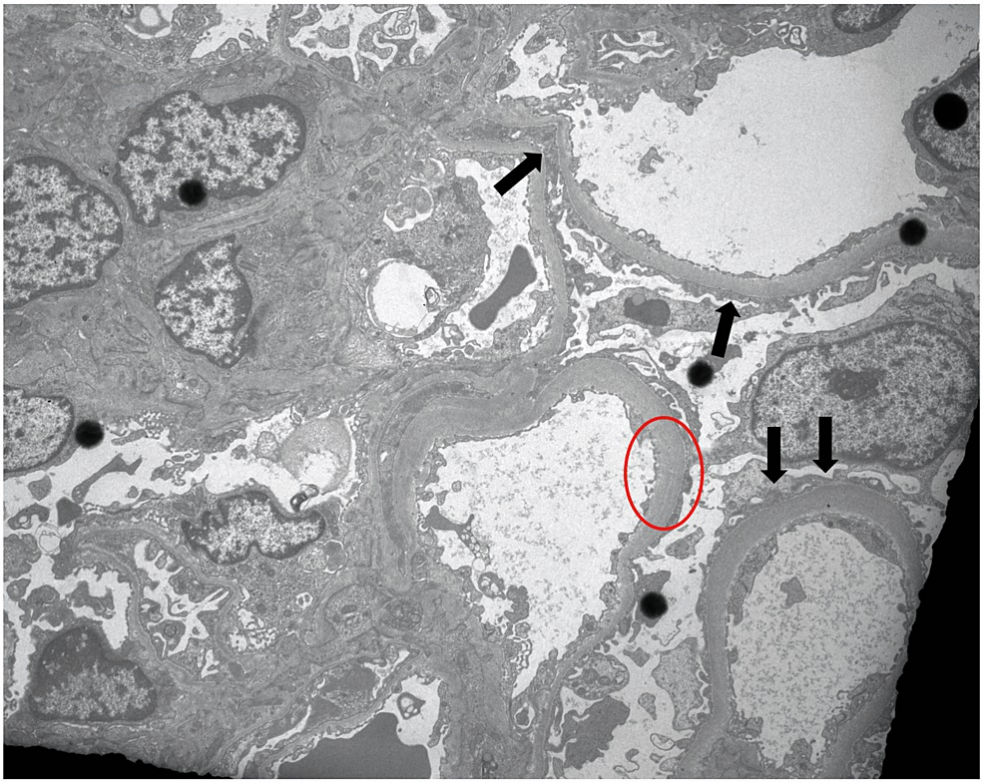
Electron microscopy with foot processes effacement (black arrows) and variable thickness of glomerular basement membrane and multilamellation (red circle).

The kidney biopsy showed swollen podocytes with no other glomerular, tubulointerstitial or vascular significant findings. Immunofluorescence was unremarkable. In the ultrastructural study there was diffuse foot processes effacement of the podocytes. The glomerular basal membrane had a variable thickness, with multilamellation of the lamina densa (Figure 1). The aspects described in the ultrastructural study raised the diagnostic possibility of Alport syndrome. A next-generation sequencing of a panel of genes associated with proteinuric nephropathies identified two heterozygous variants in the cubilin gene: c6928_6934del p.(Glu2310Cysfs*3) and c.5597C>T p. (Pro1866Leu). Parents’ genetic tests confirmed the presence of a variant in each parent and neither of them presented proteinuria to date.

The patient was started on ramipril, with a slight improvement in proteinuria (protein/creatinine ratio 1.7). After three years of follow-up, he remains asymptomatic with normal renal function (GFR 138 ml/min/1.73 m^2^).

Case 2

A 3-year-old boy was referred to the pediatric nephrology consultation due to persistent non-nephrotic proteinuria (protein/creatinine ratio 1.2), diagnosed in the context of a viral infection. He had no relevant personal history, including urinary tract infections. His mother had arterial hypertension. There were no associated complaints, namely edema, hematuria or hypertension. The urinalysis did not reveal other changes besides proteinuria. Blood analyses showed normal renal function (urea 34 mg/dL; creatinine 0.33 mg/dL), serum electrolytes, albumin (43.9 g/L) and total serum proteins (67.2 g/L). There was no anemia (Hb 13.4 g/dL) nor changes on lipid profile. The immunological study was normal (IgA, IgG, IgM, C3c, C4, ANA, anti-ds-DNA, anti-GMB antibodies, anticardiolipin antibodies). Serological studies (HIV, HCV, HBV, Epstein-Barr virus, cytomegalovirus, adenovirus, Mycoplasma pneumoniae) showed no recent infection. Renal ultrasound was normal. The DMSA scan showed two small focal areas of reduction of the renal uptake of radionuclide, one in each kidney and normal differential renal function.

**Figure 2:**
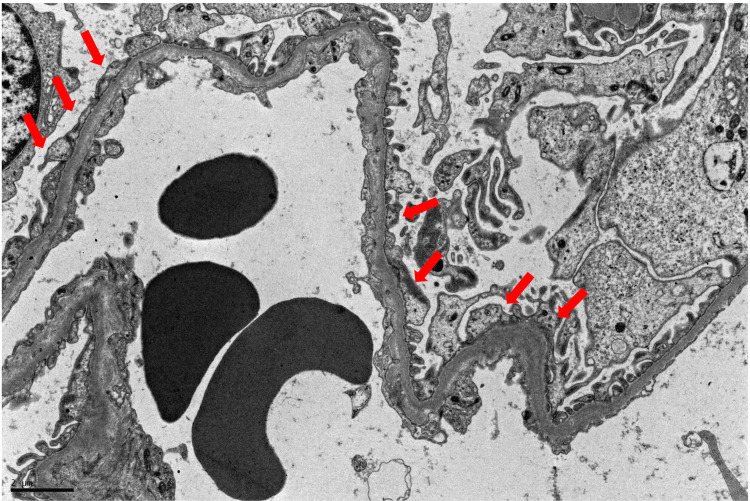
Electron microscopy with focal foot processes effacement (red arrows) and glomerular basal membrane of variable and irregular thickness.

The kidney biopsy showed swollen podocytes with no other glomerular, tubulointerstitial or vascular significant findings. In the ultrastructural study there was focal fusion of foot processes of the podocytes. The glomerular basement membranes had variable and irregular thickness (Figure 2). The aspects described in the ultrastructural study raised the diagnostic possibility of Alport syndrome. A next-generation sequencing of a panel of genes associated with proteinuric nephropathies identified two heterozygous variants in the cubilin gene: c.6007A>T p. (Arg2003*) and c.5840C>A p. (Ser1947Tyr). Parents’ genetic tests confirmed the presence of a variant in each parent, and neither of them presented proteinuria to date.

Ramipril was started with an improvement in proteinuria (protein/creatinine ratio 0.7). After six years of follow-up, the patient remains asymptomatic, with normal renal function (GFR 150 ml/min/1.73 m^2^).

## Discussion

In the literature, there are few reported cases of CUBN gene mutations associated with proteinuria. In some cases, they are associated with Imerslund-Grasbeck syndrome (IGS), an autosomal recessive disease caused by mutations in the CUBN and AMN (amnionless), which is characterized by intestinal malabsorption of vitamin B12, megaloblastic anemia and, in half of the cases, proteinuria [7]. Bedin et al. had previously described cases of CUBN gene variants associated with persistent isolated proteinuria without megaloblastic anemia, suggesting that there are different binding sites in cubilin for the vitamin B12/intrinsic factor complex and albumin, which are affected differently by the distinct mutations [7].

The two cases presented are very similar, both in terms of manifestations and evolution. Histopathology examination raised suspicion of Alport syndrome. Podocyte hypertrophy and effacement of their foot processes (usually associated with proteinuria) are common to both entities and they were already described in the literature in pediatric patients with CUBN gene mutations [6]. Other studies described minimal, unspecific or not present renal lesions on biopsies [7]. The electron microscopy result was unexpected with regard to the changes in the glomerular basement membrane (GBM), namely the irregularity of thickness and, in one case, the appearance of multilamellation of the lamina densa. These findings support the hypothesis that proteinuria in patients with CUBN gene mutations is also caused by podocyte and glomerular injury, and not only by malabsorption of the proximal tubule.

We can speculate GBM abnormalities could be the result of significant proteinuria and subsequent podocyte apoptosis or, alternatively due to primary podocyte dysfunction.

Currently, angiotensin-converting enzyme inhibitors (ACEI) and angiotensin receptor blockers (ARB) are recognized as important kidney protectors due to their anti-proteinuric, hemodynamic, anti-inflammatory and anti-proliferative effects. CUBN gene mutations patients were sometimes assumed to be non-responsive to ACEI or ARBs, but our two patients responded partially [7]. Given the glomerular damage, confirmed on renal biopsy, and their response, we decided to maintain the therapeutic.

The impact of proteinuria on cells of the nephron is uncertain, but some studies support involvement in the development of fibrosis. Accumulation of proteins in proximal tubule lysosomes, due to increased protein internalization, is thought to be associated with inflammation and fibrosis, with eventual progression to renal failure [1]. Some authors argue that the proximal tubule is able to handle a fixed daily amount of filtered albumin, but an excess can be harmful [1,8].

There are contradictory reports about CUBN gene mutations and renal outcome. Bedin et al. [7] identified 39 patients with biallelic pathogenic variants in the CUBN gene associated with persistent isolated proteinuria, with early onset in childhood. Renal function was normal in all cases. Although limited in time, the follow-up of these patients showed no progression to renal impairment. Previously in cases of IGS with proteinuria, long-term deterioration of renal function was also not described, even with the persistence of proteinuria [9,10].

On the other hand, some sporadic reports associated CUBN gene variants with progression to end-stage renal disease. A study by Reznichenko et al. [11] identified a CUBN variant as being associated with a risk of progression to end-stage renal disease and graft failure in transplanted kidneys, when the mutation is present in the donor. Quental et al. [12] described two siblings with CUBN gene mutations, diagnosed in adulthood, one of them with end-stage kidney disease as the inaugural manifestation. Domingo-Gallego et al. [13] described 15 patients with homozygous or compound heterozygous variants in CUBN genes, 13 patients with normal renal biopsy and preservation of normal kidney function and the two remaining patients with a more severe phenotype, that was associated by the authors to comorbidities.

## Conclusions

Pediatric patients with persistent isolated sub-nephrotic or nephrotic proteinuria should be submitted to blood analysis, renal biopsy and genetic test in order to clarify their diagnosis and avoid unnecessary medical treatments (for example, immunosuppression). At present, due to the uncertainty of prognosis, it is suggested to keep CUBN gene mutation patients under close surveillance of proteinuria and renal function. The variable ultrastructural patterns of podocytopathy and glomerular basal membrane alterations in kidney biopsies of pediatric patients with proteinuria should lead to the diagnostic possibility of CUBN gene mutation in the differential diagnosis.

Larger case series with longer follow-up will allow us to determine the best approach and follow-up measures, as well as the prognosis of each specific mutation in the long term.
